# Reversal of an existing hearing loss by gene activation in *Spns2* mutant mice

**DOI:** 10.1073/pnas.2307355120

**Published:** 2023-08-08

**Authors:** Elisa Martelletti, Neil J. Ingham, Karen P. Steel

**Affiliations:** ^a^Wolfson Centre for Age-Related Diseases, King’s College London, Guy’s Campus, London SE1 1UL, United Kingdom

**Keywords:** mouse genetics, reversal of disease, S1P signaling, hearing loss

## Abstract

Hearing loss is highly heterogeneous, but one common form involves a failure to maintain the local ionic environment of the sensory hair cells reflected in a reduced endocochlear potential. We used a genetic approach to ask whether this type of pathology can be reversed, using the *Spns2^tm1a^* mouse mutant known to show this defect. By activating *Spns2* gene transcription at different ages after the onset of hearing loss, we found that an existing auditory impairment can be reversed to give close to normal thresholds for an auditory brainstem response (ABR), at least at low to mid stimulus frequencies. Delaying the activation of *Spns2* led to less effective recovery of ABR thresholds, suggesting that there is a critical period for intervention. Early activation of *Spns2* not only led to improvement in auditory function but also to protection of sensory hair cells from secondary degeneration. The genetic approach we have used to establish that this type of hearing loss is in principle reversible could be extended to many other diseases using available mouse resources.

## Significance

Neurological diseases are often thought to be irreversible, including hearing loss. In this study, we found that one type of hearing loss can be reversed as long as the treatment is delivered within a critical period early in disease progression. This result is a proof of concept that hearing loss not only can be avoided but also may be reversed. This genetic approach can be used for a wide range of diseases using existing mouse resources.

Hearing impairment is very common in the population and can begin at any age. Over half of adults in their 70 s have a significant hearing loss. Hearing impairment isolates people from society, can be associated with depression and cognitive decline, and is a major predictor of dementia (1–4). The only remedies currently available are devices such as hearing aids and cochlear implants, but these do not restore normal function. There is a large unmet need for medical approaches to slow down or reverse hearing loss.

Treatment strategies for genetic deafness are being developed using the mouse, including gene suppression using siRNAs, antisense oligonucleotides to correct splicing, gene replacement, and gene editing to repair single base mutations (5–15). These studies usually involve introduction of the agent into the mouse inner ear soon after birth when the auditory system is immature, at a stage corresponding to around 16 to 24 wk of gestation in humans, making direct translation challenging. One report suggests that introduction of otoferlin (*Otof*) sequences into the mouse inner ear at later stages leads to improved hearing in *Otof* mutants that would otherwise show abnormal inner hair cell (IHC) synaptic function and deafness (16). We also are interested in asking whether an existing hearing impairment can be reversed because the main demand for treatments is from people who already have hearing loss, particularly progressive age-related hearing loss.

One significant pathological type underlying age-related hearing loss involves the maintenance of the endolymph, the high-potassium, low-sodium fluid that bathes the upper surface of sensory hair cells. In the cochlea, endolymph is maintained at a positive resting potential, the endocochlear potential (EP), of around +120 mV which is essential for normal hair cell sensitivity. We have used a genetic approach in *Spns2* mutant mice to investigate the possibility of reversing hearing loss linked to an EP deficit.

## Results

### Reversal of Hearing Loss in *Spns2* Mutants.

**Fig. 1.**
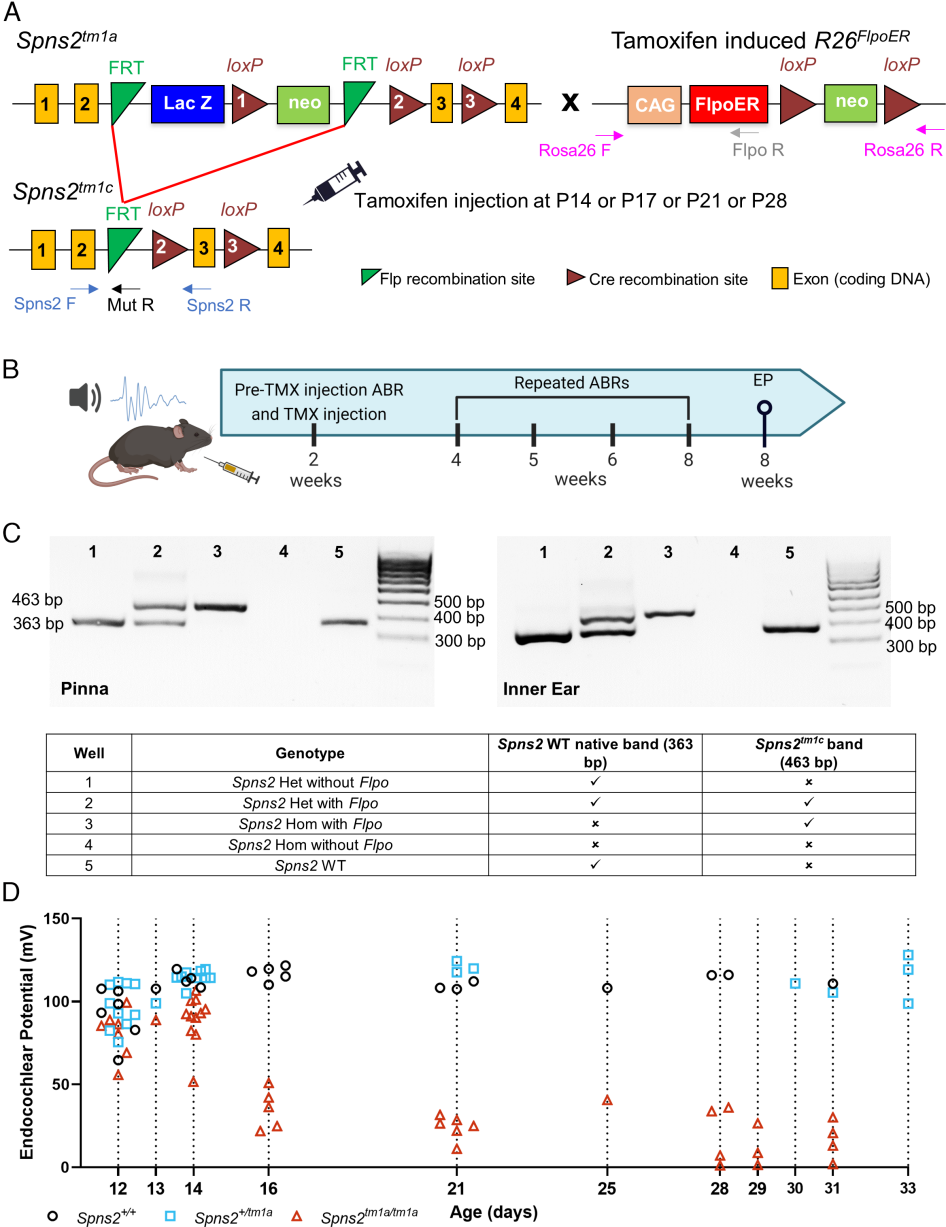
(*A*) Diagram showing the design of the *Spns2^tm1a^* and *Spns2^tm1c^* alleles. Yellow boxes show exons, green triangles show FRT sites, brown triangles show loxP sites, blue and green boxes show the neomycin resistance and *LacZ* genes, and arrows marked F and R indicate the locations of the genotyping primer sites. Upon tamoxifen injection, in the mice carrying the *R26^FlpoER^* allele, the Flpo recombinase induces recombination between the FRT sites, and the mutagenic cassette is deleted, resulting in the generation of the *Spns2^tm1c^* allele which is functional (17). (*B*) Diagram showing the experimental design of this study, using 2 wk as the starting time point as an example. ABRs were recorded at P14, P17, P21, or P28 prior to intraperitoneal tamoxifen injection (TMX, dose 0.2 mg/g). Repeated ABR tests were performed up to 8 wk old when the EP was also measured as a terminal procedure. Diagram generated in BioRender. (*C*) Agarose gel showing the PCR product of the *Spns2* F and R primers (panel *A*) from pinna (*Left*) and inner ear (*Right*) samples after tamoxifen injection. The native WT *Spns2* allele has a 363-bp PCR product, whereas the *Spns2^tm1c^* allele had a 463 bp PCR product, which is 100 bp larger due to the presence of one FRT and one loxP site. *Spns2* homozygotes without *Flpo* show no PCR product because the presence of the large DNA cassette separates the primer binding sites too far to support normal short-range PCR. (*D*) The EP was measured at different ages between P12 and P33 in control (black circles), *Spns2^+/tm1a^* (cyan squares), and *Spns2^tm1a/tm1a^* (red triangles) mice; individual mouse measurements are plotted as clusters around the dotted line indicating the age. The n of control, *Spns2^+/tm1a^*, and *Spns2^tm1a/tm1a^* mice are, respectively, as follows: at P12 6, 10, and 7; at P13 1 each; at P14 4, 9, and 11; at P16 5 for controls and *Spns2^tm1a/tm1a^*; at P21 3, 3, and 6; at P25 1 for controls and *Spns2^tm1a/tm1a^*; and at P28 to P33 4, 5, and 11. The datasets at P14, P21, and between P28 and P33 days were previously published by our group (17).

*Spns2* encodes Spinster homolog 2, a sphingosine-1-phosphate (S1P) transporter. *Spns2* mutant mice show a rapidly progressive hearing loss associated with a dramatic decline in EP between 2 and 3 wk after birth (17) (Fig. 1*D*). As EP develops to high levels at first in *Spns2* mutants (Fig. 1*D*), we considered ways of restoring it to normal levels after the onset of hearing loss. The *Spns2* mutation used is the *Spns2^tm1a^* mutation, a knockout-first, conditional-ready design that has a large DNA construct inserted into an intron that disrupts gene expression (Fig. 1*A*) (18). The construct is flanked by Flippase Recombination Target (FRT) sites that recombine on exposure to Flp recombinase, removing the construct and restoring gene function (17).

In the current study, we used a tamoxifen-inducible Flp recombinase encoded by *R26^FlpoER^* (*Flpo*) (19) to restore *Spns2* function at different stages in the disease progression. Recombination was verified in pinna skin and whole inner ear by PCR using the same primers that were used for genotyping (Fig. 1 *A* and *C*). A single intraperitoneal tamoxifen injection was given either at postnatal age (P) 14, 17, 21, or 28 d, spanning the ages immediately before (P14) and after the drop in EP (P16 onward; Fig. 1*D*). Entire litters were injected, including *Spns2^tm1a^* homozygotes, heterozygotes, and wild types both with and without *Flpo*.

**Fig. 2.**
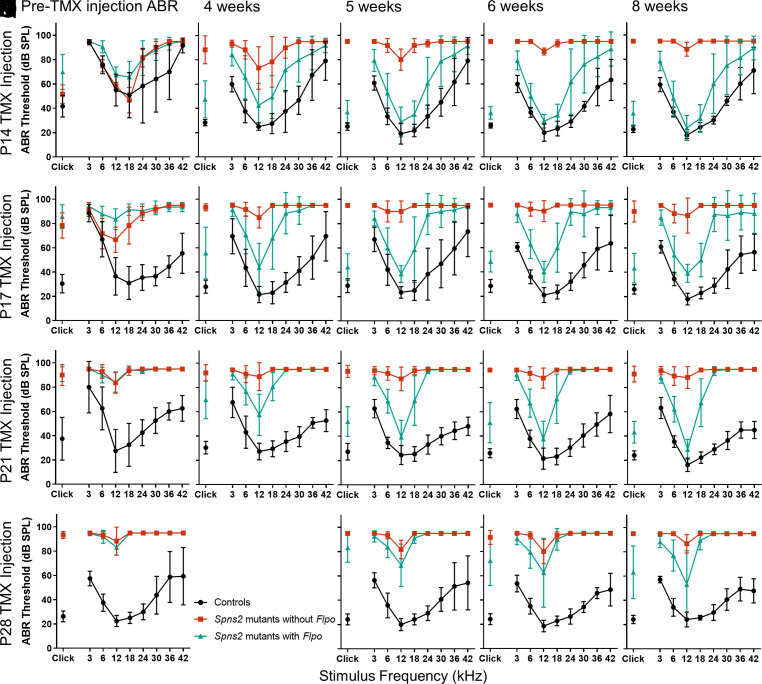
(*A*–*E*) ABR thresholds in dB SPL (Sound Pressure Level) prior to tamoxifen (TMX) injection at P14 (*A*) and following at 4, 5, 6, and 8 wk (respectively, *B*–*E*). *Spns2* mutants without *Flpo* are plotted in red (n = 3), *Spns2* mutants with *Flpo* are plotted in teal (n = 8 up to 6 wk, n = 7 at 8 wk), and their control littermates are plotted in black (n = 6 including *Spns2^+/+^* and *Spns2^+/tm1a^* mice with and without *Flpo*). *Spns2* mutants with *Flpo* show a gradual recovery in ABR thresholds at 3, 6, 12, 18, and 24 kHz in comparison with littermate *Spns2* mutants without *Flpo* which have increasing ABR thresholds across all the frequencies. No response was plotted as 95 dB SPL, the maximum sound level used. (*F*–*J*) ABR thresholds before tamoxifen injection at P17 (*F*) and after at 4, 5, 6, and 8 wk (respectively, *G*–*J*). *Spns2* mutants with *Flpo* (teal) have higher ABR thresholds at P17 than littermate controls, but after tamoxifen injection, the ABR thresholds at 6, 12, and 18 kHz improve. Controls n = 10, *Spns2* mutants without *Flpo* n = 3, *Spns2* mutants with *Flpo* n = 7 up to 6 wk, n = 6 at 8 wk. (*K*–*O*) ABR thresholds before tamoxifen injection at P21 (*K*), and after at 4, 5, 6, and 8 wk (respectively, *L*–*O*). Only the ABR thresholds at 12 kHz recovered consistently in the *Spns2* mutants with *Flpo* injected with tamoxifen at P21. At P21, controls n = 2, *Spns2* mutants without *Flpo* n = 7, and *Spns2* mutants with *Flpo* n = 7, whereas at 4, 5, 6, and 8 wk, controls n =11, *Spns2* mutants without *Flpo* n = 9, and *Spns2* mutants with *Flpo* n = 14. (*P*–*S*) ABR thresholds before tamoxifen injection at P28 (*P*) and after at 5, 6, and 8 wk (respectively, *Q*–*S*). Two *Spns2* mutants with *Flpo* failed to improve but ABR thresholds ranged between 25 and 60 dB SPL in the other six animals. Controls n = 7, *Spns2* mutants without *Flpo* n = 3, and *Spns2* mutants with *Flpo* n = 8. Data plotted as mean ± SD.

**Fig. 3.**
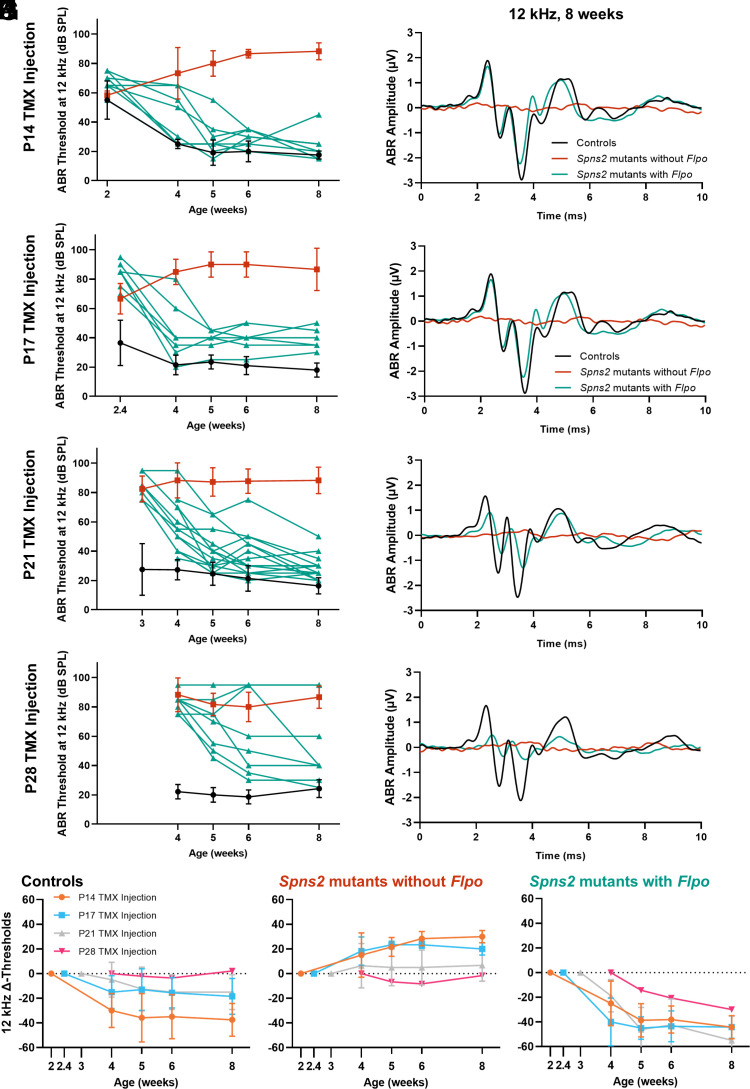
(*A*–*D*) The ABR thresholds at 12 kHz, the best-recovered frequency, are plotted against the ages when the ABRs were tested. At all four injection ages (P14 in *A*, P17 in *B*, P21 in *C*, and P28 in *D*), *Spns2* mutants with *Flpo* have lower ABR thresholds after tamoxifen exposure. In *Spns2* mutants with *Flpo* injected at P28, two out of eight mice did not recover any ABR. Controls and *Spns2* mutants without *Flpo* are plotted as mean ± SD in black and red, respectively, whereas *Spns2* mutants with *Flpo* are plotted as single animals in teal. (*E*–*G*) The mean ± SD of the change (Δ) in ABR threshold at 12 kHz compared with the preinjection threshold for each mouse is plotted with age for controls (*E*), *Spns2* mutants without *Flpo* (*F*), and *Spns2* mutants with *Flpo* (*G*). (*H*–*K*) The mean waveform evoked by a 12 kHz, 80 dB SPL stimulus is plotted for controls (black), *Spns2* mutants without *Flpo* (red), and *Spns2* mutants with *Flpo* (teal) at 8 wk old. Comparable amplitude and latency are observed between controls and *Spns2* mutants with *Flpo* in P14 injected mice. For mice injected at other ages, both reduced amplitude and longer latency are observed in the *Spns2* mutants with *Flpo* but not as severe as the *Spns2* mutants without *Flpo.* Numbers of mice are the same as for Fig. 2.

Prior to tamoxifen injection, auditory function was assessed using auditory brainstem response (ABR) measurements, and these recordings were repeated in each mouse at intervals after tamoxifen exposure until the mouse was 8 wk old, when terminal ABR and EP measurements were collected (Fig. 1*B*). Control littermates (*Spns2^tm1a^* heterozygotes or wild types with or without *Flpo*; Fig. 2, black) showed continued maturation of their thresholds from 2 to 5 wk old (Figs. 2 *A*–*C* and 3*E*), suggesting no adverse impact of tamoxifen injection. *Spns2^tm1a^* homozygotes that did not carry *Flpo* showed progressive worsening of their ABR thresholds with age (Figs. 2; red; and 3*F*). In contrast, *Spns2^tm1a^* homozygotes carrying *Flpo* showed gradual improvement in their thresholds with age (Figs. 2; teal; 3*G*), indicating reversal of their hearing loss.

### A Critical Period for Reversal of Hearing Loss.

By comparing pre- and posttamoxifen ABR thresholds in the same mouse, we observed that injection of tamoxifen at P14 led to the development of near-normal thresholds at frequencies below 24 kHz in *Spns2* mutant homozygotes carrying *Flpo* (Fig. 2 *A*–*E*). At P17, *Spns2* mutants with *Flpo* already show raised ABR thresholds (Fig. 2*F*), but after tamoxifen injection, this hearing loss was reversed at 6, 12, and 18 kHz to near-normal thresholds (Fig. 2 *F*–*J*). P21 and P28 tamoxifen injections were too late to improve thresholds for frequencies of 18 kHz or over, but for 12 kHz, some improvement was found with injection as late as P28 (Fig. 2 *K*–*S*). Comparison of the final ABR at 8 wk old of the four injected groups (Fig. 2 *E*, *J*, *O*, and *S*) showed that the earlier the activation of the *Spns2* gene, the more effective was the reversal of the hearing impairment.

Individual *Spns2* mutants with *Flpo* show raised thresholds prior to tamoxifen injection, but then, almost all of them show a continuing improvement in ABR thresholds with age at the most sensitive frequency, 12 kHz, as indicated by the downward slopes of thresholds plotted in Fig. 3 *A*–*D*. The reversal of hearing impairment was generally stable up to 8 wk old when the last ABR test was performed. The mean changes in thresholds with time compared with preinjection ABRs are shown in Fig. 3*G*, with the downward slopes showing that thresholds continued to improve up to the last age tested. ABR waveforms in *Spns2* mutants with *Flpo* are very similar to those from control littermates in the group injected at P14 suggesting effective restoration of brainstem auditory function (Fig. 3*H*), although reduced amplitudes and prolonged latencies of individual components of the waveform were observed for later ages of injection (Fig. 3 *I*–*K*).

### Rescue of EPs and Stria Vascularis Structure.

**Fig. 4.**
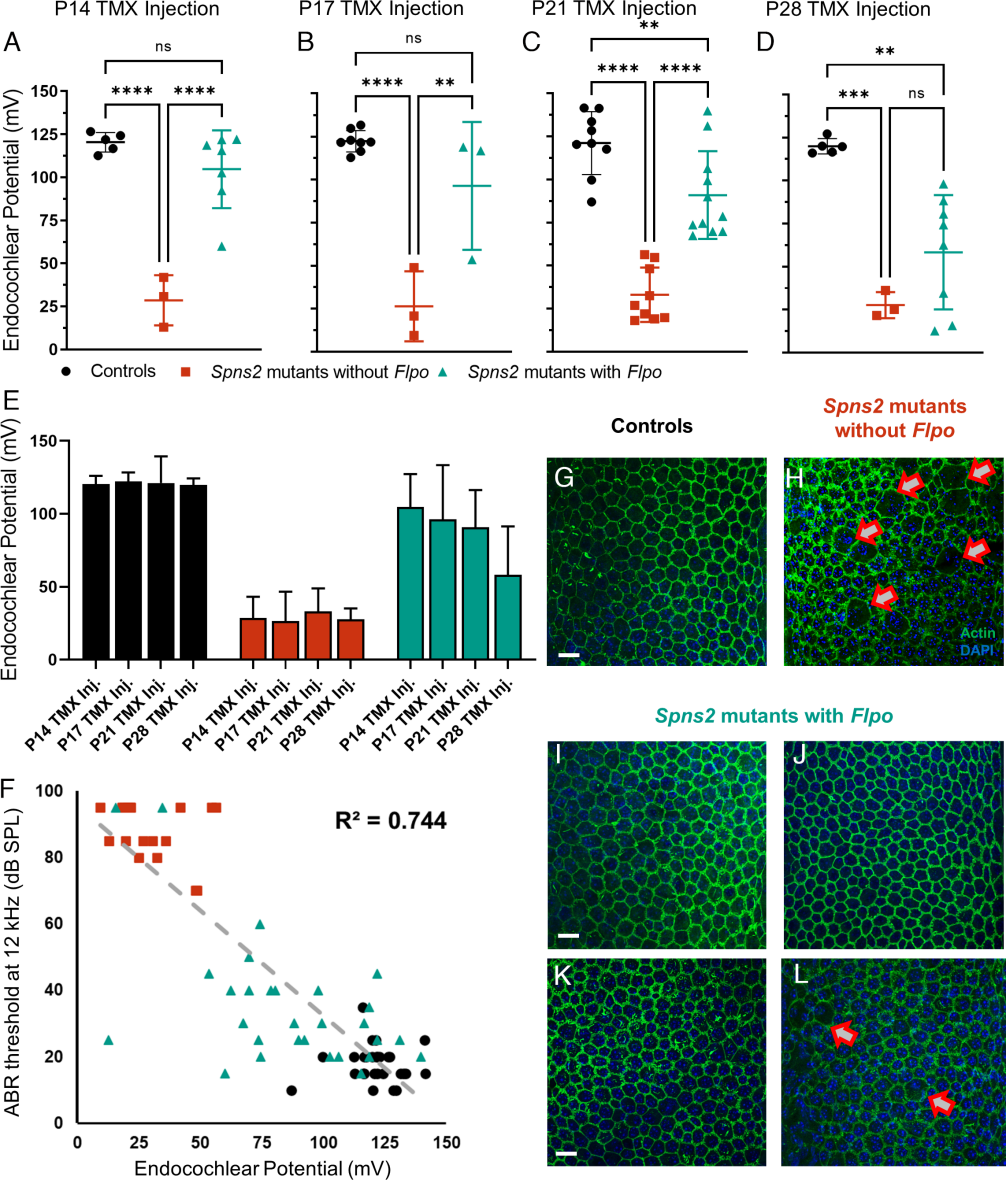
(*A*–*E*) In the mice injected at P14 (*A*), there was no significant difference in EP between the controls and *Spns2* mutants with *Flpo,* while the *Spns2* mutants without *Flpo* showed a reduced EP. Controls (n = 5) 120.4 mV ± 5.7, *Spns2* mutants without *Flpo* (n = 3) 28.6 mV ± 14.6, and *Spns2* mutants with *Flpo* (n = 7) 104.7 mV ± 22.7. In the mice injected at P17 (*B*), there was no significant difference in EP between the controls and *Spns2* mutants with *Flpo,* while the *Spns2* mutants without *Flpo* showed a reduced EP. Controls (n = 8) 122.3 mV ± 6.1, *Spns2* mutants without *Flpo* (n = 3) 26.4 mV ± 20.3, and *Spns2* mutants with *Flpo* (n = 3) 96.3 mV ± 37.1. In the mice injected at P21 (*C*), the EP is reduced in both *Spns2* mutants with *Flpo* and *Spns2* mutants without *Flpo* in comparison with littermate controls. Controls (n = 9) 121.3 mV ± 18.2, *Spns2* without *Flpo* (n = 9) 33.1 mV ± 15.8, and *Spns2* mice with *Flpo* (n = 11) 90.9 mV ± 25.4. In the mice injected at P28 (*D*), the EP was reduced in both *Spns2* mutants with *Flpo* and *Spns2* mutants without *Flpo* in comparison with littermate controls. Controls (n = 5) 119.9 mV ± 4.4, *Spns2* mutants without *Flpo* (n = 3) 27.7 mV ± 7.6, and *Spns2* mutants with *Flpo* (n = 8) 58.3 mV ± 33.2. Single mice are plotted as black circles (controls), red squares (*Spns2* mutants without *Flpo*), and teal triangles (*Spns2* mutants with *Flpo*) and as mean ± SD. One-way ANOVA test with Tukey post hoc, **P ≤ 0.01, ***P ≤ 0.001, ****P ≤ 0.0001, and ns = not significant. (*F*) With an R^2^ value of 0.744, a correlation between a lower ABR threshold at 12 kHz and higher EP was observed, recorded at 8 wk old. All of the mice with EP recordings injected at P14, P17, P21, and P28 are plotted in this graph. Controls in black circles (n = 27), *Spns2* mutants without *Flpo* in red squares (n = 18), and *Spns2* mutants with *Flpo* in teal triangles (n = 29). (*G*–*L*) Strial marginal cell boundaries show normal morphology in *Spns2* mutants with *Flpo* injected at P14 (I), P17 (*J*), and P21 (*K*) like the controls (*G*, P17 injected mice), while abnormalities were present in *Spns2* mutants without *Flpo* as indicated by the red arrows (*H*, P17 injected mice). *Spns2* mutants with *Flpo* injected at P28 showed some morphological variabilities: In two of the four samples, no obvious defects were observed; however, in one sample, the marginal cell boundaries were defective, as observed in the *Spns2* mutants without *Flpo*; and in a second sample, as shown in panel *L*, only some of the boundaries were defective. The samples used for this histological analysis were the same used for the repeated ABR and EP measurements. Actin filament (phalloidin) in green and nuclei (DAPI) in blue. (Scale bar, 20 µm.) Controls n = 4 at P14, n = 3 at P17, n = 1 at P21, and n = 2 at P28; *Spns2* mutants without *Flpo* n = 2 at P17 and n = 3 at P21; *Spns2* mutants with *Flpo* n = 4 at P14, n = 5 at P17, n = 3 at P21, and n = 4 at P28.

Measuring EP is a terminal procedure, so we cannot track EP recovery directly, unlike tracking hearing via ABR recording. Therefore, we measured EP at a single time point after the final ABR recording at 8 wk old in the tamoxifen-injected mice. EP in the control group was around +120 mV, a normal level for the mouse (Fig. 4 *A*–*E*; black). In *Spns2^tm1a^* homozygotes without *Flpo* (red), EP was severely reduced, similar to previously reported levels in these mutants (17). The EPs of *Spns2* mutants with *Flpo* (teal) injected at P14 or P17 were higher than those of *Spns2* mutants without *Flpo*, in some cases within the normal range (Fig. 4 *A*, *B*, and *E*). Injection at P21 or P28 led to lower EP measurements than earlier injection (Fig. 4 *C*–*E*).

When EP measurements at all ages of injection and all genotypes were compared with the ABR thresholds at 12 kHz of the same mice, all at 8 wk old, we found a correlation between lower ABR thresholds and higher EP with an R^2^ value of 0.744 (Fig. 4*F*).

The stria vascularis on the lateral wall of the cochlear duct generates EP by active pumping of cations into the endolymph, and in *Spns2^tm1a^* homozygotes, it shows disruption of the regular arrangement of marginal cell boundaries on the lumenal surface that is associated with reduced EP (17). We examined whole mounts of the stria vascularis from the same tamoxifen-injected mice used for ABR and EP measurements. Actin filaments at the boundaries of marginal cells were labeled using phalloidin. Strial surface preparations from *Spns2* mutants with *Flpo* injected at P14, P17, or P21 (Fig. 4 *I*–*K*) had normal morphology of the marginal cell boundaries (green) in contrast with *Spns2* mutants without *Flpo* which showed a disrupted pattern (Fig. 4*H*, red arrows indicating the abnormal marginal cells boundaries). Morphological variability was observed in *Spns2* mutants with *Flpo* injected at P28, as some samples had normal marginal cell boundaries, while others showed some defect (Fig. 4*L*).

### Does Hair Cell Degeneration Limit the Critical Period for Reversing Hearing Loss?

**Fig. 5.**
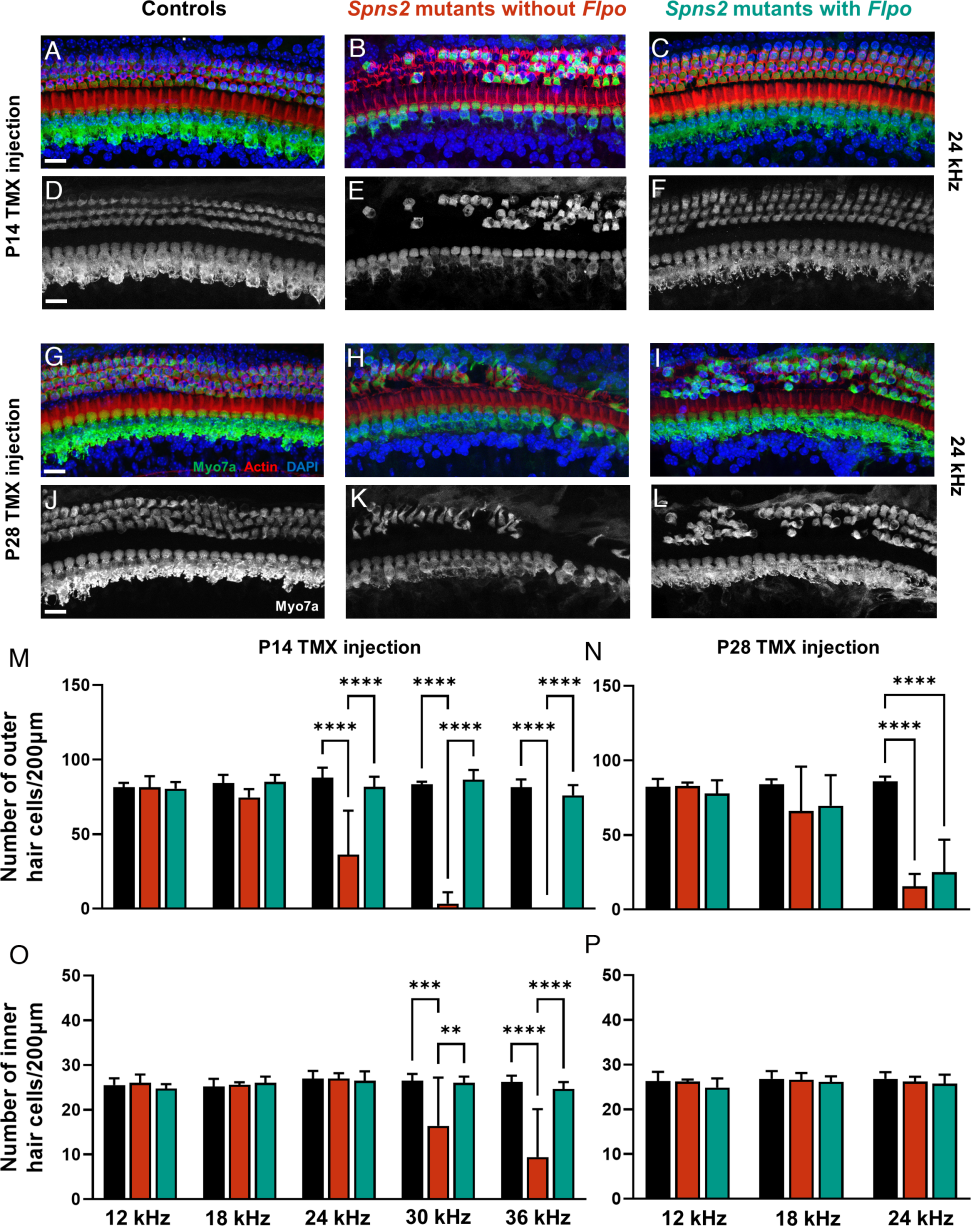
(*A*–*L*) Surface preparations of the organ of Corti at the 24 kHz best-frequency cochlear region. (*A*–*C*) and (*G*–*I*): Myo7a labels the hair cells in green, Phalloidin the stereocilia in red, and DAPI the nuclei in blue. (*D*–*F*) and (*J*–*L*) Myo7a label alone. (Scale bar, 20 µm.) (*M-P*) Quantification of hair cells remaining at different best-frequency regions. (*A*–*F*, *M*, and *O*) Tamoxifen was administered to mice at P14, and cochlear samples were collected 2 wk later, at 4 wk old. At all cochlear regions, the number of both OHCs and IHCs in *Spns2* mutants with *Flpo* (teal) is comparable to littermate controls (black). In contrast, *Spns2* mutants without *Flpo* (red) have significantly fewer OHCs at the 24, 30, and 36 kHz cochlear regions, as well as fewer IHCs at 30 and 36 kHz. Controls n = 6, *Spns2* mutants with *Flpo* n = 5, *Spns2* mutants without *Flpo* n = 2 to 5 depending on frequency region. (*G*–*L*, *N*, and *P*) Tamoxifen was administered to mice at P28, and cochlear samples were collected 2 wk later, at 6 wk old. Both *Spns2* mutants without *Flpo* and *Spns2* mutants with *Flpo* have fewer OHCs at 24 kHz in comparison with the littermate controls. Controls n = 6, *Spns2* mutants without *Flpo* n = 5, *Spns2* mutants with *Flpo* n = 7. All data are shown as mean ± SD and statistically analyzed by the one-way ANOVA test with Tukey post hoc, **P ≤ 0.01, ***P ≤ 0.001, and ****P ≤ 0.0001.

Sensory hair cell degeneration occurs in *Spns2^tm1a^* homozygotes, but this is not the cause of their hearing loss because ABR thresholds are already considerably raised before the onset of hair cell loss at around P28 (17). However, hair cell loss might contribute to the efficiency of reversal of hearing loss when *Spns2* is activated at later ages in our study. Therefore we analyzed the organ of Corti in whole mount preparations to assess hair cell loss 2 wk after tamoxifen injection at either P14 or P28. In mice injected with tamoxifen at P14, there was no significant loss of either inner or outer hair cells (OHCs) in the *Spns2* mutants carrying *Flpo* compared with the control mice (Fig. 5 *A*, *C*, *D*, *F*, *M*, and *O*; compare black with teal bars), while *Spns2* mutants without *Flpo* (red bars) showed extensive loss of hair cells in the basal turn, representing the regions most sensitive to high frequencies (Fig. 5 *B*, *E*, *M*, and *O*). Tamoxifen injection at P28 was too late to avoid OHC degeneration in the basal turn (Fig. 5 *G–L*, *N*, and *P*).

## Discussion

We found that hearing impairment due to the *Spns2* mutation can be reversed after it was first detected by activating the *Spns2* gene, and ABR thresholds improve in individual mice. Neurological disorders are often thought to be irreversible, but there have been several reports that some phenotypes can be reversed in mouse models, including Rett syndrome (20), Kabuki syndrome (21), Rubinstein–Taybi syndrome (22), *SYNGAP1* deficiency (23), and autism spectrum disorders due to *SHANK3* mutations (24). More recently, it was reported that mice with an *Otof* mutation can show improvements in ABR thresholds following gene therapy as late at 30 d after birth (16). Our finding of reversal of hearing loss in individual mice after activation of the *Spns2* gene is an important proof of concept that deafness associated with EP deficiency not only can be halted in its progression but also can be reversed. This demonstration raises the prospect that this type of hearing loss may be reversible in humans.

Very few cases have been reported so far of *SPNS2* mutations in humans. No audiometry was presented for two children who were compound heterozygotes for *SPNS2* mutations, but bilateral hearing loss with other systemic features was reported, similar to the phenotype of the *Spns2* mouse mutant (25, 26). However, genomic variants close to the *SPNS2* gene were found to be significantly associated with auditory thresholds at 4 kHz in the UK 1958 British Birth Cohort (25), suggesting a potential role for milder variants affecting this gene in hearing in the population. We studied the *Spns2* mouse mutant as an example of a mammal with reduced EP, and other examples may be helpful to establish whether different forms of strial defect might also be reversible. The genetic approach we adopted would not be directly transferable to humans, but our findings do suggest that increasing the level of transcription of the normal version of an affected gene, for example, using gene therapy, could be clinically beneficial.

Histopathology suggests that human age-related hearing loss can affect three main sites within the cochlea: sensory hair cells; synapses between hair cells and cochlear neurons; and the stria vascularis on the lateral wall of the cochlear duct (27). Analysis of human audiogram shapes compared with experimental damage to either the stria vascularis or hair cells supports the concept that strial dysfunction has a role to play in human hearing loss (28, 29). Furthermore, recent meta-analysis of genome-wide association studies of hearing loss in the human population provides additional support for the importance of strial function in the pathogenesis of hearing loss (30). Thus, this type of pathology is a good target for development of generic treatments to boost strial function.

We found that the earlier the activation of the gene, the better the restoration of cochlear function, including ABR thresholds and waveforms, EP levels, and hair cell preservation. This suggests that there is a critical period for reversing hearing loss, with the precise timing of this period depending upon the outcome measure. For example, *Spns2* activation at P17 was effective at reversing raised ABR thresholds at frequencies of 18 kHz and below but was too late to rescue thresholds at higher frequencies, 24 kHz and above. Thresholds in *Spns2* mutants with *Flpo* continue to improve up to 8 wk old, the last age we studied. The reason for this continuing improvement is not known but could be due to any of a number of steps in the process, such as slow triggering of Flp recombinase translocation; delayed recombination; ongoing increase in *Spns2* transcription; gradual alteration of S1P signaling in the target tissues; or slow buildup of stria vascularis pumping activity. The loss of hair cells when *Spns2* is activated later is likely to influence the end point of the critical period for reversing hearing loss. However, the earliest electrophysiological deficit recorded was the reduced EP (17), and we found that later activation of *Spns2* led to lower EP levels (Fig. 4 *A*–*D*). Although we cannot directly record a reversal in EP level in an individual mouse in the days following tamoxifen injection, our observations suggest that the EP can probably be increased by activating *Spns2* and that it is likely that ABR thresholds reflect this. The finding of a critical period indicates that early intervention in humans affected by the strial type of pathology would be important, before the changes leading to EP reduction become irreversible and before secondary hair cell degeneration occurs.

In summary, our finding is a proof of concept that at least one type of hearing loss can be reversed. Furthermore, the availability of over 4,000 mouse mutants carrying this design of mutation (*tm1a* or *tm1e*), many of which show abnormal phenotypes (31–33), facilitates similar investigations in other diseases that up to now have been considered irreversible.

## Materials and Methods

### Ethics Statement.

Mouse studies were carried out in accordance with UK Home Office regulations and the UK Animals (Scientific Procedures) Act of 1986 under UK Home Office licenses, and the study was approved by the King’s College London Ethical Review Committees. Mice were culled using methods approved under these licenses to minimize any possibility of suffering.

### Generation of Mutant Mice.

*Spns2^tm1a(KOMP)Wtsi^* (*Spns2^tm1a^*) mutant mice were generated at the Wellcome Trust Sanger Institute on a C57BL/6N genetic background (17, 34). B6N.129S6(Cg)-*Gt(ROSA)26Sor^tm3(CAG-flpo/ERT2)Alj^*/J mice, also known as *R26^FlpoER^* mice, express a tamoxifen-inducible, optimized FLPe recombinase variant called Flpo and were obtained from the Jackson Laboratory (19). Both mutant lines are available from public repositories ( European Mouse Mutant Archive and The Jackson Laboratory). We crossed mice carrying the *Spns2^tm1a^* and *R26^FlpoER^* alleles to generate littermates including the genotypes for analysis: *Spns2^+/+^* and *Spns2^+/tm1a^* with and without *Flpo* which formed the normal control group; *Spns2^tm1a/tm1a^* homozygotes without *Flpo*, providing the mutant group; and *Spns2^tm1a/tm1a^* homozygotes with *Flpo*, which were the experimental mice. All genotypes were injected with tamoxifen (see below).

### Genotyping.

DNA was extracted from pinna skin, and for some samples whole inner ear from 8-wk-old mice, and used as the template for short-range PCR using a common *Spns2* forward primer (5′CAAAACAATATGGGCTGGGG3′), an *Spns2* reverse primer (5′GATGAAGGCAGGACTCAGGG3′) and a mutant-specific reverse primer (5′TCGTGGTATCGTTATGCGCC3′) (Fig. 1*A*, blue and black arrows). The resulting band sizes were 363 bp for the *Spns2* wild-type (WT) product and 187 bp for the mutant product, as previously described (17). The WT product was not amplified from the *tm1a* mutant allele because the primers bound to sites that were too far apart for short-range PCR to be successful, but it can be amplified in the *tm1c* allele (Fig. 1). Upon tamoxifen injection, Flpo recombinase is translocated to the nucleus where it recognizes the FRT sites in the *Spns2^tm1a^* allele, inducing recombination with the consequent removal of the mutagenic cassette and restoration of *Spns2* gene activity. The allele generated from this recombination is called *Spns2^tm1c^*. The native WT *Spns2* allele had a 363 bp PCR product, while the *Spns2^tm1c^* allele had a 463 bp PCR product using the same primers (Fig. 1*A*), 100 bp larger due to the presence of one FRT site and one LoxP site. The presence of the *Flpo* allele was checked using the common Rosa26 forward primer 5′AAAGTCGCTCTGAGTTGTTAT3′ with either the Rosa26 reverse primer 5′GGAGCGGGGAAATGGATATG3′ or the Flpo mutant reverse primer 5′TTATGTAACGCGGAACTCCA3′ (Fig. 1*A*, magenta and gray arrows) The resulting band sizes were 603 bp for the WT product and 309 bp for the mutant product.

### Tamoxifen-Induced Gene Recombination.

Tamoxifen in corn oil (Sigma-Aldrich, C8267) was injected intraperitoneally using a single injection at a dose of 0.2 mg/g at one of four different ages: postnatal day (P)14, P17, P21, or P28 (Fig. 1*B*). ABRs were recorded the same day and before the tamoxifen injection, and then, repeated ABR tests were performed up to 8 wk old (Fig. 2) when the EP was also measured as a terminal procedure (Fig. 4 *A*–*E*). The Flpo-FRT recombination was verified on pinna or whole inner ear tissue upon DNA extraction and PCR testing using the same *Spns2* primers used for genotyping of the *Spns2* allele. Our results showed the expected recombination and generation of the *Spns2^tm1c^* allele (Fig. 1*C*).

### ABR Recording.

Mice were anesthetized by intraperitoneal injection of 100 mg/kg Ketamine (Ketaset, Fort Dodge Animal Health) and 10 mg/kg Xylazine (Rompun, Bayer Animal Health). ABRs were measured in a sound-attenuating chamber fitted with a heating blanket as previously described (25, 35). Subcutaneous recording needle electrodes (NeuroDart; Unimed Electrode Supplies Ltd.) were inserted on the vertex and overlying the left and right bullae. Responses were recorded to free-field calibrated broadband click stimuli (10 µs duration) and tone pips (5 ms duration, 1 ms onset/offset ramp, fixed phase onset) at frequencies between 3 and 42 kHz, at levels ranging from 0 to 95 dB SPL in 5 dB steps, at a rate of 42.6 stimuli per second. Evoked responses were digitized, amplified, and bandpass filtered between 300 and 3,000 Hz, and 256 responses averaged for each frequency/intensity combination to give a waveform. ABR thresholds were defined as the lowest stimulus level to evoke a visually detected waveform.

### EP Recordings.

Mice were anesthetized with intraperitoneal urethane (0.1 mL/10 g body weight of a 20% w/v solution of urethane), and EP was measured as described previously (36, 37). A reference electrode (Ag-AgCl pellet) was placed under the skin of the neck. A small hole was made in the basal turn lateral wall, and the tip of a 150-mM KCl-filled glass micropipette was inserted into the scala media. The EP was recorded as a stable positive potential compared with the reference electrode.

### Hair Cell Quantification, Lateral Wall Surface Preparation, and Confocal Imaging.

The organ of Corti was analyzed in mice injected with tamoxifen at P14 and P28 and then collected 2 wk after tamoxifen injection (Fig. 5). The lateral wall surface preparation was performed using the inner ears of the mice that were injected with tamoxifen and had the repeated ABR tests (Fig. 2) as well as the EP measurements recorded (Fig. 4). Inner ears were fixed in formaldehyde (4% w/v paraformaldehyde) for 2 h and decalcified in Ethylenediaminetetraacetic acid (EDTA) overnight at room temperature. Following fine dissection, both the lateral wall and the organ of Corti specimens were permeabilized in 5% Tween in phosphate-buffered saline (PBS) for 40 min and incubated in blocking solution (4.5 mL of 0.5% Triton X-100 in PBS and 0.5 mL of normal horse serum) for 2 h. The organ of Corti samples were incubated in Myo7a primary antibody (diluted 1:200; 25-6790, Proteus) overnight at 4 °C and then incubated for 45 min at room temperature with the secondary antibody goat anti-rabbit IgG Alexa Fluor488 (1:300, #A11008, Thermo Fisher Scientific) and phallodin (1:500, #R415, Thermo Fisher Scientific). The lateral wall samples were incubated with phalloidin for 45 min at room temperature. Both the lateral wall and organ of Corti specimens were mounted using ProLong Gold mounting media with DAPI (P36931, Life Technologies) and stored at 4 °C. Specimens were imaged using a Zeiss Imager 710 confocal microscope interfaced with ZEN 2010 software. The plan-APOCHROMAT 40× Oil DIC objective was used, and brightness and contrast were normalized for the dynamic range in all images. Z-stacks were collected at 0.5 μm, and maximum intensity projection images were generated for analysis. The best-frequency areas were determined according to the mouse tonotopic cochlear map using the ImageJ plugin “Measure Line” (38, 39). Manual quantification of both OHCs and IHCs was performed at specific frequency regions.

## Data Availability

All data are provided within the report, and the two mutant mouse lines used are available from public repositories. Previously published data were used for this work (Some published data are replotted in Fig. 1*D*, from Chen et al. (17) PMID: 25356849). All study data are included in the main text.
